# *Trypanosoma brucei* Acyl-Protein Thioesterase-like (TbAPT-L) Is a Lipase with Esterase Activity for Short and Medium-Chain Fatty Acids but Has No Depalmitoylation Activity

**DOI:** 10.3390/pathogens11111245

**Published:** 2022-10-27

**Authors:** Robert W. B. Brown, Aabha I. Sharma, Miguel Rey Villanueva, Xiaomo Li, Ouma Onguka, Leeor Zilbermintz, Helen Nguyen, Ben A. Falk, Cheryl L. Olson, Joann M. Taylor, Conrad L. Epting, Rahul S. Kathayat, Neri Amara, Bryan C. Dickinson, Matthew Bogyo, David M. Engman

**Affiliations:** 1 Departments of Pathology, Microbiology-Immunology and Pediatrics, Northwestern University, Chicago, IL 60611, USA; 2 Department of Pathology and Laboratory Medicine, Cedars-Sinai Medical Center, Los Angeles, CA 90048, USA; 3 Departments of Pathology and Microbiology and Immunology, Stanford University School of Medicine, Stanford, CA 94305, USA; 4 Department of Chemistry, The University of Chicago, Chicago, IL 60637, USA

**Keywords:** trypanosome, *Trypanosoma brucei*, post-translational modification, palmitoylation, depalmitoylation, alpha/beta hydrolase, esterase, thioesterase, lipase

## Abstract

Dynamic post-translational modifications allow the rapid, specific, and tunable regulation of protein functions in eukaryotic cells. *S*-acylation is the only reversible lipid modification of proteins, in which a fatty acid, usually palmitate, is covalently attached to a cysteine residue of a protein by a zDHHC palmitoyl acyltransferase enzyme. Depalmitoylation is required for acylation homeostasis and is catalyzed by an enzyme from the alpha/beta hydrolase family of proteins usually acyl-protein thioesterase (APT1). The enzyme responsible for depalmitoylation in *Trypanosoma brucei* parasites is currently unknown. We demonstrate depalmitoylation activity in live bloodstream and procyclic form trypanosomes sensitive to dose-dependent inhibition with the depalmitoylation inhibitor, palmostatin B. We identified a homologue of human APT1 in *Trypanosoma brucei* which we named TbAPT-like (TbAPT-L). Epitope-tagging of TbAPT-L at N- and C- termini indicated a cytoplasmic localization. Knockdown or over-expression of TbAPT-L in bloodstream forms led to robust changes in TbAPT-L mRNA and protein expression but had no effect on parasite growth *in vitro*, or cellular depalmitoylation activity. Esterase activity in cell lysates was also unchanged when TbAPT-L was modulated. Unexpectedly, recombinant TbAPT-L possesses esterase activity with specificity for short- and medium-chain fatty acid substrates, leading to the conclusion, TbAPT-L is a lipase, not a depalmitoylase.

## 1. Introduction

*S*-acylation is the only reversible protein lipidation common to all eukaryotes, comprising the addition of an acyl chain to a cysteine residue of a protein through a covalent thioester bond catalyzed by a *S*-acyltransferase enzyme; this process can be reversed through hydrolysis by an *S*-acyl thioesterase [1]. Palmitate (C16:0) is the predominant fatty acid used for *S*-acylation; hence *S*-palmitoylation or palmitoylation are synonymous names for this post-translational modification, although acyl groups other than palmitate may be involved [2,3]. *S*-acylation serves different functions depending on the protein, such as anchoring a peripheral membrane protein to intracellular membranes by kinetic trapping, targeting proteins to lipid raft microdomains, regulating the trafficking of proteins to intracellular membranes, stabilizing proteins to prevent ubiquitination and degradation, and directly regulating the activity of membrane receptors and ion transporters [1,4,5]. Hundreds of palmitoylated proteins with diverse cellular functions have been identified in many cell types, using proteomics to define each unique palmitoyl proteome (palmitoylome), although there is no strict amino acid consensus sequence for palmitoylation [6,7,8,9].

*S*-acyltransferases with multiple transmembrane domains and an Asp-His-His-Cys (DHHC) cysteine-rich domain (CRD) required for auto- and trans-palmitoylation were first identified in yeast [10,11]. Subsequently, twenty-four DHHC proteins were found in humans, which are also referred to as zinc-finger DHHC domain (zDHHC or zfDHHC) proteins or palmitoyl acyltransferases (PATs). They are predominately found in the Golgi apparatus or endoplasmic reticulum (ER), but also in the plasma membrane and yeast vacuole [12,13]. DHHC proteins have different preferences for acyl chains of different lengths as well as specific substrates, although there can be functional redundancy [3].

In contrast, protein deacylation depends on several distinct enzymes having thioesterase activity that belong to the α/β-hydrolase fold superfamily of proteins. They hydrolyze the covalent thioester bond to release palmitoyl-CoA (or another acyl group) [14,15,16,17,18]. Palmitoyl protein thioesterase (PPT1) was the first depalmitoylase discovered in eukaryotes [19]. However, acyl protein thioesterase 1 (APT1) is the predominant cytoplasmic depalmitoylase in eukaryotes, and APT2 is another depalmitoylase homologue with a substrate specificity different from APT1 [20,21,22,23]. All these depalmitoylase enzymes possess the S-H-D catalytic triad and a G-X-S-X-G consensus motif as determined by their crystal structures [15,16,24]. Depalmitoylases enable dynamic *S*-acylation cycles as a mechanism to spatially organize peripheral membrane proteins, whereby proteins palmitoylated in the Golgi apparatus are trafficked along the secretory pathway to the plasma membrane; proteins that mislocalize to endomembranes are depalmitoylated by APTs and returned by retrograde transport to the Golgi so they can be repalmitoylated and trafficked properly to their intended destinations [25,26]. Remarkably, APTs are themselves regulated by dynamic *S*-acylation, being palmitoylated at the Golgi, before autodepalmitoylating, so they can participate in the *S*-acylation cycle to depalmitoylate mislocalized proteins [27].

We previously identified twelve putative PATs in the genome of the protozoan parasite *Trypanosoma brucei*, as well as 124 high confidence palmitoylated proteins in procyclic forms (PF) of the parasite, but depalmitoylation is still unexplored in trypanosomes and no thioesterase has been identified [28]. In this study, we report the identification and characterization of an alpha/beta hydrolase domain (ABHD) homologue of human APT1 in *T. brucei* which we name *T. brucei* APT-like (TbAPT-L). Surprisingly, this enzyme has no thioesterase activity, and instead it catalyzes the hydrolysis of short and medium-chain fatty acid ester substrates.

## 2. Materials and Methods

### 2.1. Bioinformatics Analysis and Multiple Sequence Alignment (MSA)

Sequences used in this study were obtained from the TriTrypDB database [29]. The NCBI protein database was used for *S. cerevisiae* APT1, and the human APT1 and APT2 sequences [30]. The ToxoDB database was used for the *Toxoplasma gondii* sequence. The amino acid sequences were trimmed and aligned using Clustal Omega online algorithm and shaded with ExPASy BoxShade webtool so that amino acids across all four sequences with 100% identity and 100% similarity are highlighted in black and grey, respectively, [31].

### 2.2. Cell Culture

All *T. brucei* cell lines generated for this study were derived from the 29–13 PF cell line or the BF “single marker” cell line, both were originally derived from Lister strain 427, and were engineered to co-express bacteriophage T7 RNA polymerase and tetracycline (TET) repressor to permit TET-inducible transcription [32]. PF parasites were cultured at 27 °C in SDM-79 medium supplemented with 10% dialyzed FBS (Sigma-Aldrich, Saint Louis, MO, USA), 7.5 μg/mL hemin, 100 U/mL penicillin/streptomycin, 15 μg/mL G418 and 50 μg/mL hygromycin [33]. BF parasites were cultured at 37 °C with 5% CO_2_ in HMI-9 medium supplemented with 10% FBS, 10% serum plus medium complement (SAFC Biosciences, Lenexa, KS, USA), 100 U/mL penicillin/streptomycin, and 2.5 μg/mL G418 [33]. Inducible RNAi and over-expression cell lines were cultured under continuous drug selection with 2.5 μg/mL phleomycin. Cell density was measured in duplicates manually for each culture for each experiment using a Neubauer hemocytometer.

### 2.3. Alamar Blue Cell Viability Assay

1000 cells/well were incubated in HMI-9 medium for 48 h with an optimized range of palmostatin B or 2-BP concentrations in triplicate. The cell viability dye Alamar Blue was added at 44 h and incubated for 4 h so a change in fluorescence could be measured at 520 nm emission and 545 nm excitation on a FLUOstar Omega spectrophotometer (BMG Labtech GmbH, Ortenberg, Germany) [34]. All data points were normalized to the HMI-9 medium control wells for relative fluorescence. A non-linear variable slope four parameter curve fit was used to calculate IC50 values.

### 2.4. Depalmitoylation Probe (DPP) Fluorescence Assays in Live Cells

Twenty million SM427 BF or PF cells were incubated in HMI-9 or SDM-79, respectively, with 0, 4, 10 or 20 μM palmostatin B for 30 min. Cells were washed twice with, and resuspended in, phosphate-buffered saline (PBS) with 60 mM glucose and 20 mM glycerol (optimized to ensure sufficient substrates for their survival for duration of experiment). Five million cells were aliquoted in triplicate in 96-well optical non-treated flat-bottom black plate (Thermo Scientific Nunc #265301) and incubated with 0, 4, 10, or 20 μM palmostatin B and 2 μM DPP-5 fluorescent substrate for 150 min. Readings were taken every minute (excitation at 490 nm and emission at 545 nm, gain = 500) using the BMG Labtech FLUOstar Omega spectrophotometer [35].

### 2.5. DNA Construct Cloning and Transfections

All primers used in the study are listed in Table 1. For the generation of TbAPT-L RNAi mutants in PF *T. brucei*, a unique 443 bp region in the middle of the TbAPT-L coding sequence (Tb927.8.6390), identified by RNAit software, was amplified from 29–13 genomic DNA (Primers 1 + 2) [36]. The PCR product was cloned into PCR BLUNTII-TOPO Vector (Invitrogen, Carlsbad, CA, USA), sequenced, digested and subcloned into the pZJM RNAi vector [37]. For the generation of TbAPT-L over-expression cell lines, the coding sequence minus the stop codon (840 bp) of TbAPT-L was amplified from genomic DNA (Primers 7+8), restriction digested with 5′ *Hind*III and 3′ *Xba*I and cloned into TET-inducible pLEW79-NOG1-2 × myc vector (gift of Marilyn Parsons). Alternatively, the full coding sequence (843 bp) was amplified (Primers 9 + 10), restriction digested with 5′ *Xho*I and 3′ *Bam*HI and cloned into TET-inducible eYFP-pLEW100v5 vector (gift of George Cross).

Transfection of BF parasites was performed using AMAXA Nucleofector^®^ II (Lonza, Allendale, NJ, USA). For each transfection 5 × 10^7^ cells were centrifuged at 500× *g*, the pellets resuspended in PBS with 13 mM glucose (PBSg), centrifuged and washed twice, and resuspended in 100 μL Amaxa Human T-cell solution (Lonza Cat. No. VPA-1002) at the concentration of 5 × 10^8^ cells/mL in an aluminum cuvette [38]. DNA was prepared for transfection by linearization with *Not*I, gel extraction, and ethanol precipitation. 10 μg of linearized TbAPT-L pZJM, TbAPT-L-2×myc pLEW79, eYFP-TbAPT-L pLEW100v5, or water control, were added to the cells resuspended in T-cell solution and transfected using Program X-001. Cells were transferred to fresh HMI-9 medium and plated at two different serial dilutions on 24-well plates and left to recover at 37 °C. Antibiotic selection was initiated 16 h post-transfection by the addition of drug corresponding to the construct (2.5–5.0 μg/mL phleomycin for pZJM, pLEW100v5, and pLEW79). RNAi induction was initiated with the addition of 1 μg/mL TET. 

Transfection of PF parasites was performed using the BIO-RAD Gene Pulser™ electroporator (BIO-RAD, Hercules, CA, USA). DNA was prepared for transfection by linearization with *Not*I, gel extraction, and ethanol precipitation. For each transfection 2 × 10^7^ cells were centrifuged at 1000× *g*, the pellets resuspended in PBSg, centrifuged, washed twice, and resuspended in 0.53 mL electroporation medium (120 mM KCl, 0.15 mM CaCl_2_, 9.2 mM K_2_HPO_4_, 25 mM HEPES, 2 mM EDTA, 4.75 mM MgCl_2_, 69 mM sucrose, pH 7.6) at ~4 × 10^7^ cells/mL. Cells were transferred to a 0.4 cm gap electroporation cuvette (BIO-RAD Cat. No. 165-2088), 10 μg of linearized TbAPT-L pZJM, TbAPT-L-2 × myc pLEW79, or water control, were added to the cells, and electroporated twice at 1.5 kV, 25 μF with 10 s between pulses. Cells were transferred to fresh SDM-79 medium, and antibiotic selection was initiated 16 h post-transfection by the addition of drug corresponding to the construct (2.5 μg/mL phleomycin for pZJM and pLEW79).

### 2.6. Immunofluorescence Microscopy

PF cells were pelleted by centrifugation at 1000× *g* for 10 min, washed twice in PBSg, allowed to settle onto poly-L-lysine coated slides for 15 min and extracted with ice-cold 1% Triton X-100 on ice. Slides were fixed for 30 min on ice in 4% paraformaldehyde prepared in PBS, and fixative was quenched with 50 mM glycine for 12 min. Fixed cells were then treated with 0.2% Triton X-100 prepared in PBS for 5 min for cell permeabilization. After a few washes, slides were incubated in blocking buffer (2% normal goat serum, 1% bovine serum albumin (BSA) in PBS) for 30 min and incubated overnight at 4 °C with calflagin-specific rabbit serum at 1:500, 9E10 anti-myc mouse serum at 1:10 or PFR2 specific rabbit serum (kindly provided by Hill lab, UCLA) at 1:1500 in blocking buffer, followed by a 30-minute wash with cold PBS. Slides were then incubated for 1 h in Alexa Fluor 488-conjugated goat anti-rabbit secondary antibody or Alexa Fluor 568-conjugated goat anti-mouse secondary antibody (Life Technologies, Carlsbad, CA, USA) diluted at 1:500 in blocking buffer and washed for 30 min with PBS. After a quick wash with water, slides were dried and mounted in Prolong Gold Antifade Mountant with 6-diamidino-2-phenylindole (DAPI) (Life Technologies, Carlsbad, CA, USA). Imaging of TbAPT-L-2×myc was performed using a Zeiss Axio Imager Z1 (Peabody, MA, USA) using a 100× objective. Image acquisition and de-convolution were performed using ZenPro 2.3 software (Carl Zeiss Microscopy GmbH, Jena, Germany). 

BF cells are less adherent to glass slides than PF cells, so the protocol was modified accordingly. Cell density was counted and 5 × 10^6^ cells were centrifuged at 700× *g* for 4 min. The supernatant was removed, and the cell pellet was resuspended in 1 mL 1× PBSg as a wash step to remove residual serum. The centrifugation and wash steps were repeated, and the final pellet was resuspended in 400 μL 4% paraformaldehyde for fixation. Cells were settled onto the poly-L-lysine coated wells of slides for 1 h and extracted with −20 °C cold methanol overnight in −20 °C freezer. The fixative was quenched with 0.2% glycine for 3 × 10 min. The wells were incubated with blocking buffer (2% normal goat serum, 1% BSA in PBS) overnight. Cells were incubated for 1 h at room temperature with mouse anti-calflagin polyclonal serum at 1:2000 dilution in blocking buffer, followed by a 3 × 10 min washes with cold PBS + 0.05% Tween 20 (PBS-T). Slides were then incubated for 1 h in goat anti-mouse Alexa Fluor 568-conjugated secondary antibody (Life Technologies, Carlsbad, CA, USA) diluted at 1:2000 in blocking buffer and washed for 3 × 10 min with PBS-T. Excess liquid was aspirated and slides were mounted with Prolong Glass Antifade Mountant with NucBlue™ (Life Technologies, Carlsbad, CA, USA) and sealed cover slips. Imaging of YFP-TbAPT-L was performed using a Zeiss Axio Imager Z2 with Apotome for structured illumination using a 100× objective lens and Zen 2.3 Pro software for z-stack acquisition set for 0.24 μM slices and compressed with the orthogonal projection function.

### 2.7. In Vitro Growth Curves

WT, TbAPT-L RNAi, and TbAPT-L-2 × myc BF cell lines were seeded at an initial density of 5 × 10^4^ cells/mL in two flasks, and one culture of each was induced by the addition of 1 μg/mL TET. Cell density was measured in duplicates manually for each culture daily using a Neubauer hemocytometer. Cultures were diluted to 5 × 10^4^ cells/mL with fresh medium every day to maintain parasites in the logarithmic phase of growth. The growth rate factor for each day was calculated and the previous cell density was multiplied by that factor every day to create a theoretical cell density without dilution. The experiment was repeated (n = 2) and the mean and S.E. values for the cumulative growth curve were plotted in GraphPad Prism version 8 (San Diego, CA, USA).

### 2.8. qRT-PCR Detection of TbAPT-L Expression from cDNA

RNA was isolated from cell pellets using TRIzol™ reagent (ThermoFisher Scientific, Waltham, MA, USA) following the manufacturer’s protocol. A minimum of 10 million trypanosomes from each culture were required for an adequate yield of RNA to make cDNA. Cells were centrifuged at 500× *g* for 10 min and supernatant removed before the addition of 1 mL TRIzol™ reagent. Isolated RNA was treated with Ambion TURBO DNA-free DNase I kit (ThermoFisher Scientific) to remove contaminating DNA. cDNA was prepared from total RNA using Maxima H minus cDNA synthesis kit (ThermoFisher Scientific), as described by the manufacturer. Expression of TbAPT-L was determined by quantitative real-time PCR (qRT-PCR). Reactions were set up in 10 μL final volume with: 1× PowerUp™ SYBR™ Green Master Mix (ThermoFisher Scientific), 0.5 μM final concentration of primers pairs, and 1 μL of the cDNA template diluted 1 in 4. Primers 3+4 were used to amplify an 81 bp amplicon within the TbAPT-L coding sequence outside the region targeted by RNAi and Primers 5+6 were used to amplify a 108 bp amplicon within the housekeeping gene telomerase reverse transcriptase (TERT) as a control (Table 1). Thermal cycling was performed by the QuantStudio 5 qRT-PCR machine (ThermoFisher Scientific) in the following steps: 1. UDG activation: 50 °C for 2 min; 2. Dual-Lock DNA polymerase: 95 °C for 2 min; 3. Denature: 95 °C for 15 s; 4. Annealing: primer TM: 60.0 °C for 1 min; 5. Repeat steps 3–4 for 40 cycles; 6. Melt curve (dissociation stage): 95.0 °C for 15 s; 60.0 °C for 60 s; 95 °C for 15 s. Analysis of relative gene expression was calculated using the 2^−ΔΔCT^ method and TbAPT-L C_T_ values were normalized to TERT C_T_ values [39]. The results from two experiments (n = 2) were used to calculate the mean and S.E. values for relative expression and were plotted in GraphPad Prism version 8.

### 2.9. Recombinant Protein Expression and Purification

For generation of a construct to produce recombinant TbAPT-L protein, the full-length TbAPT-L coding sequence (843 bp) was amplified (Primers 11 + 12), restriction digested with 5′ *Nde*I and 3′ *Xho*I, and cloned into pET-28a(+) vector (Novagen, Madison, WI, USA). Site-directed mutagenesis of TbAPT-L ORF was performed using QuikChange II Site-Directed Mutagenesis kit (Agilent, Santa Clara, CA, USA) to mutate its catalytic serine 171 to alanine (ΔS171A) (Primers 13 + 14). Sanger sequencing confirmed the mutation was correct (Primers 15 and 16 in separate reactions). 6× His-TbAPT-L and 6× His-TbAPT-L-ΔS171A expression was induced in *E. coli* BL-21 strain with 1 mM IPTG for 3 h. Bacterial lysates in binding buffer (500 mM NaCl_2_, 20 mM Tris-HCl, pH 7.9) were purified using nickel-nitrilotriacetic acid (Ni-NTA) affinity purification column (GE healthcare), washed in buffer, and eluted in 250 mM imidazole elution buffer, followed by overnight dialysis in Tris-buffered saline (50 mM Tris, 150 mM NaCl, pH 7.6). 

### 2.10. Western Blotting and Detection of TbAPT-L Protein from Whole Cell Lysates

SDS PAGE was performed with crude whole cell lysates (5 × 10^6^ cell equivalents) from days 1 and 10 for all six cultures. Western blotting was performed with a wet transfer apparatus onto a PVDF membrane, and the conditions for washes and antibody (Ab) incubations recommended by the LI-COR Biosciences protocol were followed. The two experimental repeats were blotted separately. TbAPT-L (30 kDa) was detected with 1:1000 mouse anti-TbAPT-L polyclonal primary Ab and 1:20,000 LI-COR goat anti-mouse IRDye 800 secondary Ab; and subsequently re-probed without stripping for detection of β-tubulin (50 kDa) with 1:1000 mouse anti-beta tubulin E7 monoclonal Ab (Developmental Studies Hybridoma Bank at the University of Iowa) and 1:20,000 LI-COR goat anti-mouse IRDye 680 secondary Ab. A LI-COR Biosciences Odyssey CLx was used for fluorescence band detection. Relative band intensity was calculated using LI-COR Image Studio function to measure densitometry of bands normalizing TbAPT-L to β-tubulin bands and setting WT -TET as 1.00 for both days 1 and 10 and correcting all other samples from each timepoint to that reference. 

### 2.11. Nitrophenyl Octanoate Assay

Esterase activity was investigated in *T. brucei* cell lysates in a biochemical assay using 4-nitrophenyl octanoate (4-NPO) as the substrate [40,41]. SM427 BF WT, TbAPT-L KD or OE cell lines were incubated with or without TET for 72 h prior to the assay. 50 million cells were washed twice at 500× *g* for 10 min with PBSg. The cell pellets were frozen, and subsequently resuspended in lysis buffer (20 mM HEPES, 150 mM NaCl) on ice, and sonicated at 4 °C using a pre-cooled Fisherbrand™ Q500 with recirculating chiller #4900 (QSonica, Newtown, CT, USA) and cup horn #431C2 at 50% amplitude with a 10 s pulse and 20 s pause 18 times. The sonicated lysates were centrifuged at 16,000× *g* for 30 min, and the supernatant was harvested. A bicinchoninic acid (BCA) assay was performed to determine the protein concentration of the lysates. 30 μg of each lysate was pre-incubated with or without 20 μM palmostatin B for 30 min at 37 °C. An emulsion of 120 mM 4-NPO was prepared in HEPES NaCl buffer with 10% (*v*/*v*) Triton X-100 by vigorous stirring and subsequent dilution in buffer to give an emulsion with 3 mM 4-NPO and 0.25% (*v*/*v*) Triton X-100. Esterase activity was measured in 10 μg of cell lysates in triplicate using 600 μM of the 4-NPO substrate (Sigma-Aldrich, Milwaukee, WI, USA) in the presence or absence of 20 μM palmostatin B in 96-well plate format and readings were taken every minute for 60 min (absorbance at 401 nm, gain = 500) using the BMG Labtech FLUOstar Omega spectrophotometer. The negative controls were 4-NPO with or without palmostatin B, but no cells, and these values were subtracted as background from the readings of the lysates. Three biological repeats of the assay were performed, and the results combined (n = 3).

### 2.12. Fluorogenic Substrate Assays

The quenched substrate for thioesters (QStE) probe was dissolved in DMSO. Assays were set up in a 96-well well optical non-treated flat-bottom black plate (Thermo Scientific Nunc #265301) in 100 μL final volume with substrate at 10 μM final concentration in quadruplicates [22]. Human APT1, APT2, *T. gondii* ASH1, trypsin, and TbAPT-L recombinant protein were diluted with QStE buffer (20 mM HEPES, 150 mM NaCl, 10 mM CHAPS, pH 7.4) to 50 nM, 150 nM, 100 nM, 100 nM, and 500 nM, respectively. Fluorescence (λex = 410 nm and λem = 450 nm) was read at 37 °C every 60 s using a Cytation 3 imaging reader (BioTek, Winooski, VT, USA) for 60 min. TbAPT-L recombinant protein had no thioesterase activity.

4-methylumbelliferone (4MU) fluorogenic esterase substrates with different length acyl chains were dissolved in DMSO (1 mM): acetate (2:0), butyrate (4:0), heptanoate (7:0), octanoate (8:0), decanoate (10:0), palmitate (16:0), stearate (18:0). Assays were set up in a 96-well plate with substrate at 10 μM final concentration in quadruplicates [42]. TbAPT-L recombinant protein was added at 150 nM final concentration. Fluorescence (λex = 365 nm and λem = 455 nm) was read at 37 °C every 60 s using a Cytation 3 imaging reader for 70 min. Three biological repeats were performed (n = 3). Relative fluorescence was calculated compared to DMSO only control and the mean and S.E. values were plotted in GraphPad Prism version 8.

## 3. Results

### 3.1. Trypanosoma brucei Cells Possess a Depalmitoylation Activity Sensitive to Palmostatin B

**Figure 1:**
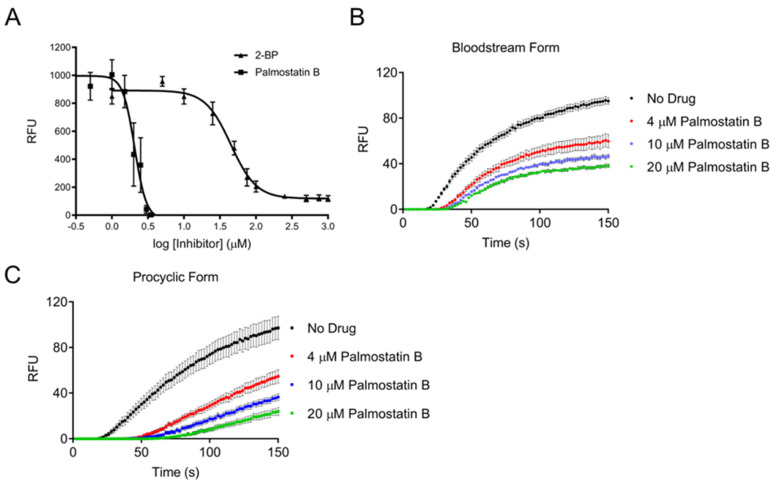
Depalmitoylation activity is essential in live *T. brucei* bloodstream (BF) and procyclic form (PF) cells. (**A**) Alamar blue assay of *T. brucei* BF cells shows dose response inhibition of cell viability by palmostatin B and 2-bromopalmitate (2-BP). Palmostatin B inhibits depalmitoylation and 2-BP inhibits palmitoylation (positive control). A non-linear variable slope four parameter curve fit was used to calculate IC_50_ values for palmostatin B (2.1 μM; n = 3) and 2-BP (44.8 μM; n = 4). (**B**,**C**) Depalmitoylation activity in live *T. brucei* BF and PF cells is sensitive to palmostatin B as demonstrated by a fluorescence assay using DPP-5 substrate, with relative fluorescence units (RFU) plotted on the y axis. Five million cells were aliquoted in triplicate in 96-well plate format and incubated with 0, 4, 10, or 20 μM palmostatin B and 2 μM DPP-5 fluorescent substrate for 150 min. Readings were taken every minute (excitation at 490 nm and emission at 545 nm, gain = 500) and plotted at two min points here for clarity of graphical representation.

Our previous studies established palmitoylation as an abundant post-translational modification in trypanosomes, but depalmitoylation has never been experimentally confirmed, although enzymatic removal of acyl groups is required for a dynamic *S*-acylation cycle [28]. Therefore, we first incubated trypanosomes with palmostatin B known to inhibit human APT1 and disrupt depalmitoylation [41]. The cell viability of bloodstream form (BF) trypanosomes was measured after 48 h of incubation with increasing concentrations of palmostatin B to generate a dose-inhibition curve (Figure 1A) [34]. The *S*-palmitoylation inhibitor 2-bromopalmitate (2-BP) was included as a positive control [43]. BF parasites were sensitive to palmostatin B with an IC_50_ of 2.1 μM, which is comparable to the value for human recombinant APT1 (670 nM) [41]. We previously calculated the IC_50_ for 2-BP for BF cells as 226 μM by cell counting, but the current value was lower (44.8 μM), reflecting differences in the assay methodology; sampling across the inhibitory dose range was better in the current study, thus improving the curve fit [28]. Both palmostatin B and 2-BP have off-target effects; 2-BP inhibits multiple proteins including human APT1 [44,45]. Palmostatin B is a pan-depalmitoylase inhibitor of human FASN, PNPLA6, ABHD6, ABHD16A, and ABHD17A/B/C [18,46]. A cell viability assay was also performed using ML348, an inhibitor specific for APT1, but it was not possible to generate an inhibition curve, as Alamar Blue fluorescence was only minimally reduced at the highest concentration at which the drug was soluble [15]. These results show that a palmostatin B sensitive activity is required for trypanosome survival, but the parasites are resistant to ML348, suggesting that their complement of depalmitoylases may be different from other eukaryotes, or may be structurally different. 

Masked fluorogenic probes which mimic APT substrates have recently been developed to measure thioesterase activity in live cells. The “turn-on” depalmitoylation probes (DPPs) are thioacylated peptide-based molecules that release a fluorophore when hydrolyzed by an S-deacylase [47,48,49]. We found that both DPP-2 and DPP-5 fluorogenic probes detect thioesterase activity in trypanosomes, despite their slightly different chemical composition. However, DPP-5 is more physiologically relevant for investigating *S*-depalmitoylation as it has a cysteine *S*-palmitoyl substrate for direct reporting of this activity, whereas DPP-2 has an octanoyl lipid moiety. *T. brucei* BF cells (Figure 1B) and PF cells (Figure 1C) were incubated in their normal media for 30 min with 0, 4, 10, and 20 μM palmostatin B, washed and resuspended in PBS supplemented with glucose and glycerol to ensure cell survival. Cells were incubated with the DPP-5 probe for 150 min at 37 °C in the presence of different concentrations of palmostatin B. Fluorescence emission was detectable after a lag phase of 20–30 min, perhaps due to slow uptake into the parasites, then increased linearly in both life cycle stages, before gradually saturating (Figure 1B,C). This activity was sensitive to palmostatin B in a dose-dependent manner, and 20 μM was insufficient to totally inhibit fluorescence emission under these assay conditions. No change in fluorescence of DPP-5 occurred without cells. These results provide the first experimental evidence that depalmitoylation is essential trypanosomes. 

### 3.2. Identification of α/β Hydrolases and APT1 Homologue in Trypanosoma brucei 

Bioinformatics identified candidate proteins in trypanosomes for further biochemical validation of depalmitoylation activity. APTs comprise a highly conserved class of enzymes that belong to the α/β hydrolase family and contain both an S-H-D catalytic triad and a G-X-S-X-G motif centered on a catalytic serine residue [19,50]. The arrangement of the nucleophile-histidine-acid catalytic triad always occurs in that order in the primary sequence and the catalytic nucleophile is typically either Ser, Cys, or Asp, and the acid is Asp or Glu [51,52]. The TriTrypDB genome database for *T. brucei* strain TREU927 was searched for proteins containing α/β hydrolase domains using text searches and InterPro domain accession numbers [29,51,53]. Fifty-five proteins contained an alpha/beta hydrolase domain (IPR0290580) – of these, seven contained the alpha/beta hydrolase fold-1 domain (IPR000073), one protein contained the alpha/beta hydrolase fold-3 domain (IPR013094), ten proteins contained a fungal lipase-like domain (IPR002921), and one protein contained the phospholipase-carboxylesterase-thioesterase domain (IPR003140). 

In addition, reciprocal BLASTp searches of the TREU927 strain genome were performed using the amino acid sequences of human APT1 (NP_ 006321) and APT2 (NP_009191), as well as yeast APT1 (NP_013219), and the apicomplexan parasite *Toxoplasma gondii* PPT1/ASH1 (TGME49_028290) [40,54]. In all searches a putative lysophospholipase gene (Tb927.8.6390) was the only significant hit, which could also find the original query in the human, yeast, or *T. gondii* genomes. Interestingly, two highly divergent homologues of human ABHD17A/B/C (NP_112490, NP_057098, NP_067037) with conserved S-H-D residues were also identified in the TREU927 strain by reciprocal BLASTp (Tb927.9.10970 and Tb927.1.4780) [18,55]. These two trypanosome genes are also homologues of *T. gondii* ASH2 (TGME49_054690) and ASH3/ASH4 (TGME49_023510 and TGME49_062490) identified in additional searches (Tb927.9.10970 and Tb927.1.4780, respectively) [54]. However, the BLASTp search for a homologue of human PPT1 did not yield any hits [19]. 

**Figure 2:**
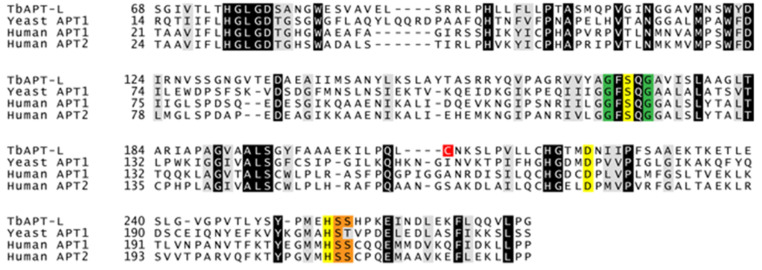
Multiple sequence alignment of TbAPT-L (Tb927.8.6390) with human and yeast homologues. The human APT1 (NP_006321.1) and APT2 (NP_036072.1) and yeast APT1 (NP_013219) amino acid sequences were used to search the TriTrypDB database by BLASTp, identifying Tb927.8.6390 as the top hit in all searches, which was confirmed by reciprocal BLASTp of this sequence in the NCBI database to find the original search queries as top hits. The amino acid sequences were trimmed and aligned using Clustal Omega online algorithm and shaded with ExPASy BoxShade webtool so that amino acids across all four sequences with 100% identity and 100% similarity are highlighted in black and grey respectively. The residues of the catalytic SDH triad (S119, D174, H208) from human APT1 are highlighted in yellow: serine nucleophile, acidic aspartic acid residue; and histidine residue, and are conserved in TbAPT-L (S171, D223, H255). The glycine residues of the G-X-S-X-G motif centered on the catalytic serine are also conserved across homologues (green). The serine residues (S209, S210) adjacent to the histidine catalytic residue in human APT1 are phosphorylated and also conserved in TbAPT-L (S256, S257) (orange). A cysteine residue (C210) (red) is predicted to be palmitoylated in TbAPT-L by the CSS-Palm 4.0 algorithm.

Given that APT1 is the canonical depalmitoylase in other eukaryotes, we hypothesized that the homologue Tb927.8.6390 would be functionally equivalent in trypanosomes. We initially referred to this protein as TbAPT1, but results from studies described below led us to refer to it as TbAPT-like (TbAPT-L). It is the second ABHD gene investigated in trypanosomes, the first being PFC19 (Tb927.10.10140) [56]. Homologues of TbAPT-L are found in all kinetoplastids in the genome database, and the N-terminus contains a conserved stretch of approximately 60 amino acids that neither aligns with other eukaryotic APT sequences, nor possesses any conserved domain hit in eukaryotes. The multiple sequence alignment of TbAPT-L with yeast APT1, and human APT1 and APT2 is trimmed for this reason (Figure 2). Only 37 residues are identical between these homologues, but the S-H-D catalytic triad residues and a G-X-S-X-G motif found in human APT1 (S119, D174, H208) are conserved in TbAPT-L (S171, D223, H255) [16]. Two serine residues phosphorylated in human APT1 (S209 and S210) are conserved in TbAPT-L (S256, S257) [57]. In addition, cysteine 210 is a predicted palmitoylation site in TbAPT-L using CSS-Palm 4.0 [58]. A structure was predicted for TbAPT-L by homology modeling with its amino acid sequence using SWISS-MODEL (data not shown), and the topology of α-helices and β-sheets and position of catalytic motifs are conserved between HsAPT2 and TbAPT-L. 

We also explored what is currently known about the TbAPT-L gene in the metadata deposited in TriTrypDB from next generation sequencing studies. The ribosome profiling dataset revealed that TbAPT-L has a higher translation level in BF than PF parasites (96th percentile in BF vs. 61st percentile in PF [59]. Consequently, our experiments described below were primarily performed in the infective BF life cycle stage.

### 3.3. TbAPT-L Is a Soluble Enzyme with Cytoplasmic Localization

**Figure 3:**
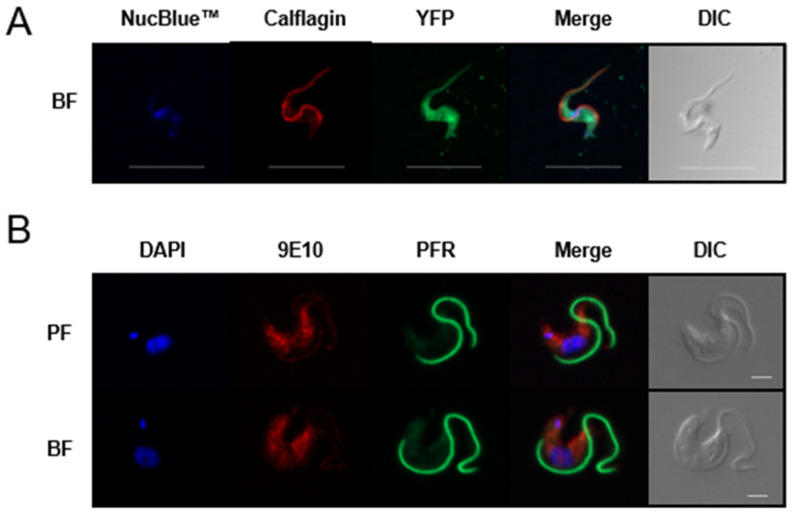
TbAPT-L is a soluble protein with diffuse localization in the cytoplasm. Immunofluorescence microscopy of N- and C-terminal tagged TbAPT-L protein in paraformaldehyde fixed trypanosomes. (**A**) Native fluorescence of YFP-TbAPT-L in bloodstream form (BF) parasites (green) together with immunofluorescence staining for flagellar calflagin (red) and nucleic acid staining with NucBlue™ (blue). Scale bar: 10 µm. (**B**) TbABDH1-2×myc in both procyclic form (PF) and BF life cycle stages detected with myc-specific 9E10 (red), flagellum-specific anti-paraflagellar rod protein 2 (green), and nucleic acid marker DAPI (blue). Scale bar: 2 µm.

Epitope-tagging of TbAPT-L was performed to determine its localization in parasites. Plasmids for both N- and C-terminal tagging were used because signal peptides, other targeting sequences, and post-translational modifications, are often located at either end of proteins, which can be disrupted by these tags. Furthermore, native proteolytic processing can unexpectedly remove them. The TbAPT-L coding sequence was cloned into two tetracycline (TET)-inducible over-expression (OE) plasmids to generate a yellow fluorescent protein (YFP)-TbAPT-L fusion protein or a TbAPT-L-2×myc fusion protein. Transfectants expressing each plasmid were produced using standard methods. Live cell imaging of the YFP-TbAPT-L expresser revealed strong native fluorescence after induction (data not shown). Paraformaldehyde (PFA)-fixed cells retained native fluorescence with YFP-TbAPT-L found throughout the cell body (Figure 3A). Expression of TbAPT-L-2×myc was confirmed by Western blotting (not shown) and the protein was detected in the cytoplasm (Figure 3B). Previous studies on human APT1 reported its palmitoylation and localization in the cytoplasm, plasma membrane, Golgi, and mitochondrion [27,35,60,61].

### 3.4. TbAPT-L Is Dispensable for In Vitro Growth in Bloodstream

**Figure 4:**
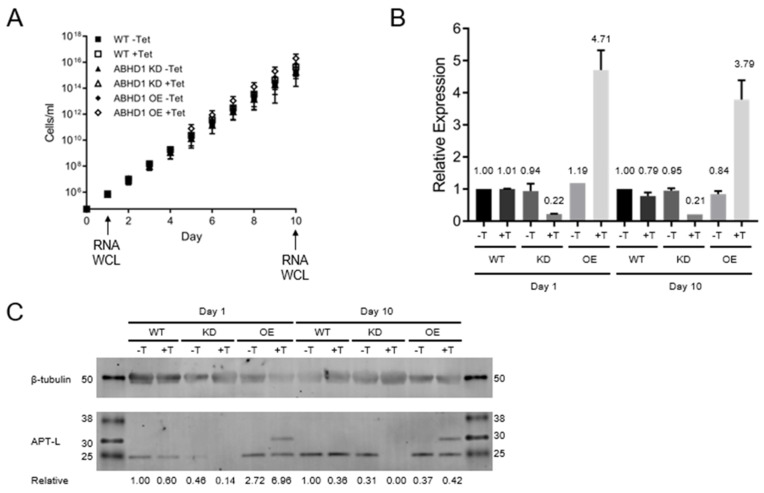
TbAPT-L is not essential for growth in vitro. (**A**) Cumulative growth curve (n = 2) for 10 days with *T. brucei* SM427 bloodstream form (BF) cells lines: wild type (WT), TbAPT-L pZJM (KD), and TbAPT-L plew79-2×myc (OE), with or without 1 μg/mL tetracycline (-T or +T). RNA and whole cell lysate were prepared at days 1 and 10 for analysis. (**B**) qRT-PCR analysis of APT-L expression at days 1 and 10 normalized to the housekeeping gene telomerase reverse transcriptase (TERT) as a control. Relative quantification values are noted above each bar. (**C**) Western blot analysis of whole cell lysates for TbAPT-L (25 and 30 kDa) β-tubulin (50 kDa) at days 1 and 10 from all cells and conditions (see above). Note that the TbAPT-L protein runs at a smaller size (25 kDa) in cell lysates than for the fusion protein (OE lanes). Relative band intensity was calculated by densitometry normalizing TbAPT-L to β-tubulin bands and setting WT -Tet as 1.00 for both days 1 and 10 and correcting all other samples from each timepoint to that reference.

To further evaluate the function of TbAPT-L we generated cells with reduced protein levels via tetracycline (TET)-induced, RNAi-mediated knockdown (KD), or producing excess protein by TET-induced overexpression (OE) [37]. The log phase growth of the KD and OE cell lines was compared with wild type (WT) cells by measuring cell density every 24 h for 10 days. There was no difference in the combined cumulative growth curves over 10 days of any of these cell lines, indicating TbAPT-L KD or OE has no effect on growth in vitro (Figure 4A). 

For validation, cells were harvested for RNA and protein analysis at 24 and 240 h post-TET-induction for analysis. mRNA levels were determined by quantitative real-time PCR (qRT-PCR) [62]. All values at each time point were normalized to the WT -TET sample. At 24 h, RNAi of TbAPT-L reduced mRNA to an average of 22% of WT control and remained stable at 21% after 10 days (Figure 4B), while TbAPT-L OE increased TbAPT-L mRNA to 471% of WT, which was again stable at 379% after 10 days. The mRNA levels of TbAPT-L remained consistently close to WT control for the transgenic cell lines in the absence of TET, indicating tight control of T7 RNA polymerase expression by the TET repressor. 

A corresponding decrease in the TbAPT-L protein level was confirmed using polyclonal mouse serum specific for recombinant TbAPT-L (Figure 4C). Western blot analysis revealed that TbAPT-L protein expression in KD cells was 14% of WT after 24 h of TET induction and was undetectable after 10 days. The TbAPT-L protein of 25 kDa was smaller than the expected 30 kDa, suggesting post-translational processing. An additional protein of 30 kDa (TbAPT-L-2×myc) was present in the OE cells. The double myc tag epitope is predicted to increase the protein by 2.4 kDa so the >5 kDa size difference between endogenous TbAPT-L and TbAPT-L-2×myc on the blot suggests that the tag protects against the presumed proteolytic processing at the C-terminus. The relative changes in protein levels were not as consistent as those of the mRNA, which is commonly observed in trypanosomes (Figure 4C). 

These results provide evidence that TbAPT-L is dispensable for the *in vitro* growth of BF parasites. In addition, preliminary results from the PF cell lines made with these identical constructs showed no difference in growth after TET induction, although they were not rigorously studied (data not shown).

### 3.5. Depalmitoylation Activity in Live Bloodstream form Cells Is Independent of TbAPT-L Expression

**Figure 5:**
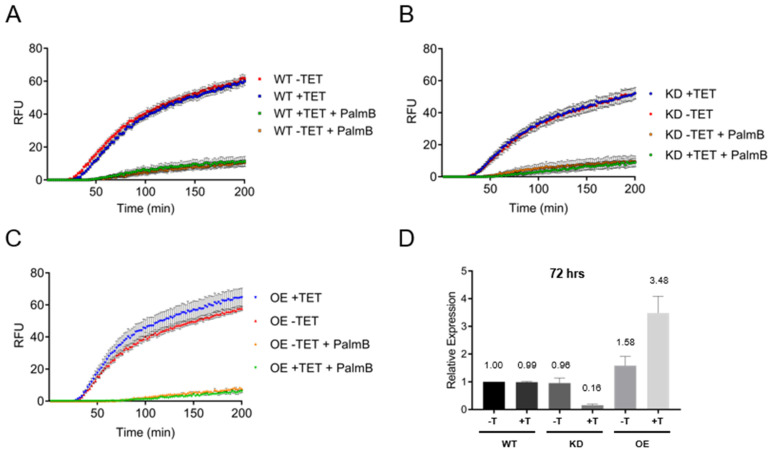
TbAPT-L is not responsible for depalmitoylation activity in live bloodstream form cells. SM427 bloodstream form (BF) wild type (WT), APT-L knockdown (KD) or over-expression (OE) cell lines were incubated with or without tetracycline (TET). Depalmitoylation activity was measured in 5 million cells in triplicate using 2 µM of the fluorescent probe DPP-5 in the presence or absence of 10 µM PalmB, and readings were taken every minute for 200 min (excitation at 490 nm and emission at 545 nm, gain = 500). The negative controls were DPP-5 with or without PalmB, but no cells. The figures represent three biological repeats (n = 3) and standard error is plotted as dotted lines. (**A**) Depalmitoylation activity of WT cells with or without TET and inhibited with PalmB had significantly reduced depalmitoylation activity as reported in Figure 1B. (**B**) Depalmitoylation activity of APT-L KD cells was not significantly different with or without TET, with similar kinetics to WT cells, although activity was slightly lower in this cell line. (**C**) Depalmitoylation activity of APT-L OE cells was not significantly different with or without TET, with similar kinetics to WT cells. (**D**) TbAPT-L mRNA expression was reliably regulated by TET induction in these live cells for this assay as reported in Figure 4B and Figure 5D. qRT-PCR analysis was performed on cells at 24- and 72-h post-induction. Abbreviation: relative fluorescent units (RFU).

WT, TbAPT-L KD and TbAPT-L OE cell lines were incubated with or without TET for 72 h, and a depalmitoylation assay was performed on these live cells using the DPP-5 depalmitoylase substrate ± palmostatin B. Cells were also harvested at 72 h for analysis of TbAPT-L mRNA levels. The results confirmed that the assay kinetics were identical for the WT cells -TET or +TET and ± palmostatin B, as expected (Figure 5A). Given our original hypothesis that TbAPT-L was the functional homologue of APT1 from other eukaryotes, we expected to see a reduction in depalmitoylation upon TbAPT-L inhibition. Surprisingly, there was no difference between the fluorescence curves of TbAPT-L KD -TET or +TET (Figure 5B), although the maximum RFU was slightly lower than WT cells (52 vs. ~60); TbAPT-L KD -TET or +TET mRNA levels were 96% and 16% of WT-TET, respectively (Figure 5D). Similarly, there was no significant difference between the fluorescence curves of TbAPT-L OE -TET or +TET (Figure 5C), and TbAPT-L OE -TET or +TET mRNA levels relative to WT-TET were 158% and 348%, respectively (Figure 5D). Therefore, these results suggest that TbAPT-L does not contribute to the palmostatin B-sensitive depalmitoylation activity detected in BF parasites with the DPP-5 substrate. We should note that the overall amino acid sequence of TbAPT-L is poorly conserved with those of APT1 homologues from other eukaryotes (Figure 2), so synthetic substrates may not bind to the active site with the same affinity, even if the S-H-D triad is conserved. 

### 3.6. Esterase Activity in Bloodstream form Lysates Is Independent of TbAPT-L Expression

**Figure 6:**
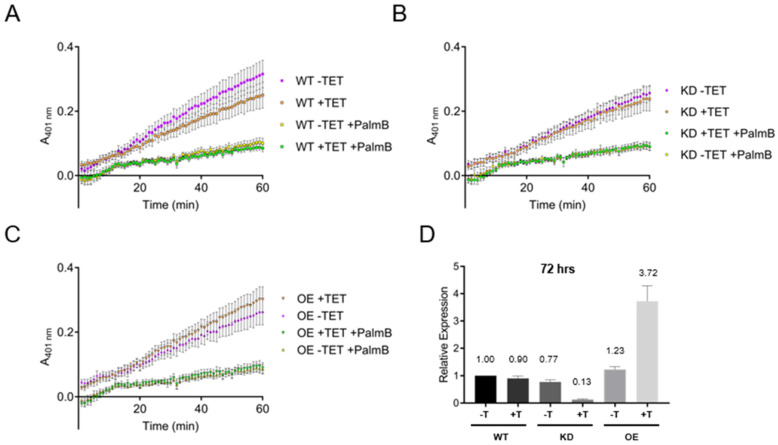
Alteration of TbAPT-L expression does not change total cellular esterase activity of lysates. SM427 BF bloodstream form (BF) wild type (WT), APT-L knockdown (KD) or over-expression (OE) cell lines were incubated with or without tetracycline (TET). Esterase activity was measured in triplicate using 600 µM of the 4-nitrophenyl octanoate (4-NPO) substrate with or without 20 µM Palmostatin B (PalmB), and A401 readings were taken every minute for 60 min. The negative controls were 4-NPO with or without PalmB, but no cells, and these values were subtracted as background from the readings of the lysates. The figures represent three biological replicates (n = 3) and standard error is plotted as bar lines. (**A**) Esterase activity in *T. brucei* WT bloodstream form lysates was partially inhibited by 20 µM PalmB. (**B**) Esterase activity in APT-L KD cells -TET or +TET did not differ significantly, with similar kinetics to WT cells. (**C**) Esterase activity of APT-L OE cells -TET or +TET was not significantly different with or without TET, with similar kinetics to WT cells. (**D**) TbAPT-L mRNA expression was reliably regulated by TET induction in these cell lines used to make lysates for this assay as reported in Figure 4B. qRT-PCR analysis was performed on cells at 24- and 72-h post-induction for each of the three biological repeats (n = 3).

Consequently, we decided to test for broad-specificity esterase activity using 4-nitrophenyl octanoate (4-NPO) substrate, which is readily hydrolyzed by other APT1 homologues [40,41]. As for the DPP-5 assays, WT, TbAPT-L KD, and TbAPT-L OE cell lines were incubated with or without TET for 72 h, and cells were also harvested for RNA. It was not possible to assay live cells using 4-NPO, so cells were sonicated on ice to produce lysates. Protein concentrations were normalized, and 30 μg of each lysate was pre-incubated with 0 or 20 μM palmostatin B for 30 min. Lysates were then incubated with the 4-NPO probe for 60 min with 0 or 20 μM palmostatin B, and A401 readings were taken every minute. The results were equally unambiguous – absorbance increased linearly over time, but there was no difference in the absorbance curves for WT -TET or +TET (Figure 6A), TbAPT-L KD -TET or +TET (Figure 6B), or TbAPT-L OE -TET or +TET (Figure 6C), despite robust changes in the mRNA levels of TbAPT-L KD -TET or +TET (77% and 13%) and TbABDH1 OE -TET or +TET (123% and 372%) relative to WT -TET. Therefore, BF lysates show esterase activity detectable by the 4-NPO substrate which is sensitive to palmostatin B, but TbAPT-L does not contribute to that activity. One explanation for this could be the functional redundancy of esterase activity by multiple trypanosome enzymes. We studied the effects of TbAPT-L KD in a mouse model of acute *T. brucei* infection and found that TbAPT-L depletion had no effect on parasitemia (data not shown). 

### 3.7. TbAPT-L Is a Bona Fide α/β Hydrolase with Esterase Activity for Synthetic Fluorogenic Short and Medium Acyl Chain Esters

**Figure 7:**
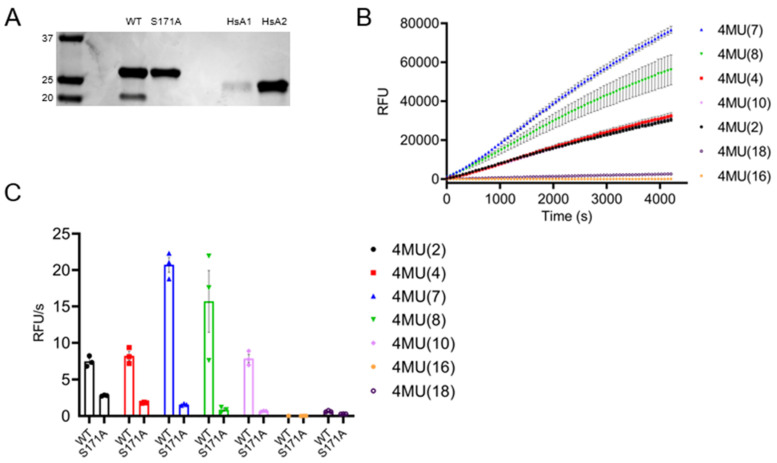
TbAPT-L has a preference for mid-length synthetic fluorescent acyl ester substrates but not palmitoyl esters. TbAPT-L is an α/β hydrolase dependent on serine 171 for its catalytic activity. (**A**) Nickel-NTA affinity purified recombinant 6×His-TbAPT-L (WT) and mutant 6×His-TbAPT-L-ΔS171A (S171A) proteins (both 32 kDa) from *E. coli* cell lysates compared to human APT1 (HsA1) and APT2 (HsA2) proteins (both 25 kDa) on a Coomassie Blue stained SDS PAGE gel. (**B**) A fluorescence assay was used to test TbAPT-L-mediated hydrolysis of seven separate 4-methyllumbelliferone (4MU) ester substrates to determine esterase activity and specificity by measuring relative fluorescence units (RFU) per second. Recombinant TbAPT-L hydrolytic activity increases for 4MU substrates with longer acyl chains until its activity peaks for heptanoate, above which its activity decreases with an increase in acyl chain length. (**C**) Rate of fluorescence change per second comparing TbAPT-L WT with the TbAPT-L-S171A mutant with each 4MU substrate measured in triplicate. The rate of hydrolysis was calculated by using the linear phase of the progress curve (initial velocity). This esterase activity is dependent on the active site serine, as the TbAPT-L-ΔS171A mutant has significantly reduced activity for all substrates. The figures represent three biological repeats (n = 3) and the mean of each is plotted with standard error.

Because TbAPT-L proved not to be a depalmitoylase, we decided to test whether at a minimum it possessed esterase activity as measured by the 4-NPO assay. Recombinant TbAPT-L, a mutant TbAPT-L in which the putative catalytic serine was mutated to alanine (TbAPT-L-S171A) and two human APTs (HsAPT1 and HsAPT2), were tested (Figure 7A). TbAPT-L possessed only one-fiftieth the activity the HsAPT1 and HsAPT2, although TbAPT-L-S171A completely inert in this assay (data not shown). These results are concordant with the 4-NPO assay results showing no difference in activity between the lysates from TbAPT-L KD or OE cell lines, as the activity for this substrate is very low. Thioesterase activity was assayed with a fluorescent quenched substrate for thioesters (QstE), but there was no detectable fluorescence (data not shown).

Subsequently, we tested the hydrolysis of seven different fluorogenic 4-methyllumbelliferone (4MU) ester substrates using a fluorescence assay to determine substrate specificity. Hydrolysis of the substrates by TbAPT-L released the fluorescent moiety over time, with preference for substrates with fatty acid chain lengths C7:0 > C8:0 > C4:0 > C10:0 > C2:0 (Figure 7B). This hydrolysis was significantly reduced by the S171A mutation (Figure 7C). The failure of TbAPT-L to hydrolyze C16:0 is consistent with the results from the TbAPT-L KD and OE live cell depalmitoylation assays employing the DPP-5 substrate that possesses a palmitoylated cysteine residue with a methyl-amide modification (Figure 5). Therefore, the results of these assays with different chemistries provide persuasive evidence that TbAPT-L cannot hydrolyze ester or thioester linked palmitoyl chains but does hydrolyze short and medium chain fatty acid esters.

## 4. Discussion

The goals of our study were to test for depalmitoylation activity in trypanosomes and to identify the depalmitoylase enzyme(s) responsible for this activity. We detected depalmitoylation in live trypanosome parasites for the first time using fluorescent substrates that was inhibited by palmostatin B (Figure 1A–C). We also identified a candidate depalmitoylase by bioinformatics – a homologue of human APT1 containing an S-H-D catalytic triad and G-X-S-X-G motif (Figure 2) Surprisingly, TbAPT-L exhibited no depalmitoylation activity in live cells (Figure 5A–D). Rather it is cytoplasmic (Figure 3A,B) lipase with preference for cleaving short and medium chain fatty acid esters (Figure 7A,B). Through the 4-NPO assay, there appears to be a functional redundancy of enzymes with esterase activity (Figure 6A–D). Therefore, TbAPT-L is dispensable for growth in BF parasites (Figure 4A–C) and a mouse model of infection (data not shown). These results imply depalmitoylation is catalyzed by another enzyme in trypanosomes, but first the limitations of our study need to be discussed.

RNAi is convenient but mRNA ablation is never 100% complete, whereas making a stable knockout (KO) cell line takes a long time and is not always possible if the gene is essential. The RNAi silencing of TbAPT-L in BF did not cause a noticeable growth phenotype in vitro over ten days (Figure 4A). This was surprising as we hypothesized that TbAPT-L functioned as a depalmitoylase and inhibition of depalmitoylation with palmostatin B caused growth inhibition (Figure 1A). Indeed, the knockdown was robust—protein expression was undetectable on day 10 (Figure 4C), even though TbAPT-L mRNA expression was only reduced to ~20% (Figure 4B). A TbAPT-L KO cell line would have been preferable for this investigation, since even a small amount of enzyme with a high catalytic rate could confound the comparison with WT parasites. However, robust TbAPT-L OE had no effect on growth, so we doubt that total TbAPT-L protein ablation by KO would have led to a growth defect, more likely there is functional redundancy from other lipases.

The unaltered depalmitoylation activity in the live KD and OE cell lines was unexpected as TbAPT-L is the only APT homologue identified in *T. brucei* genome (Figure 5A–C). These assays were performed independently of the growth curve experiments, and mRNA expression was reduced to similar levels, so we are confident in these findings (Figure 5D). Similarly, esterase activity in lysates detected by the 4-NPO assay was unaltered upon modulating TbAPT-1 expression (Figure 6A–D). Therefore, recombinant protein assays were essential to understand substrate preferences of the enzyme, although there was no detectable thioesterase activity with QStE fluorescent substrates [22]. However, the 4-NPO assay did demonstrate that TbAPT-L has low esterase activity, and we confirmed the necessity of the predicted nucleophilic serine (S171) for esterase activity with the fluorogenic 4MU ester substrates (Figure 7B,C). 

The substrate preference of TbAPT-L for short and medium-chain fatty acids but not palmitoylated proteins is reminiscent of human lysophospholipase-like 1 (LYPLAL1) which shows preference for short-chain substrates [63]. LYPLAL1 is a homologue of APT1, and TbAPT-L is a low confidence homologue. The LYPLAL1 crystal structure showed that the rear-end of the hydrophobic tunnel in its active site is closed to long-chain fatty acids on palmitoylated protein substrates by bulky residues compared to human APT1 [63]. Indeed, given the similarity to APT1 (LYPLA1) and LYPLAL1, TbAPT-L could possess lysophospholipase activity for cleaving the sn-1 acyl group of lysophospholipids including lysophosphatidylcholine (LPC) [64]. Similarly, human PPT2 has broader range of thioesterase activity against medium-chain fatty acid substrates (C8:0 to C:20) than PPT1, which has narrow optimal activity for palmitoyl-CoA and palmitoylated protein substrates, but PPT2 cannot bind palmitoyl cysteine or palmitoylated proteins due to structural differences in the lipid binding groove [65]. Therefore, determining the structure of TbAPT-L would be useful to understand its function. 

In addition, many other ABHD protein family members are lipases with short chain ester hydrolase activity and the G-X-S-X-G motif, such as monoacylglycerol lipase 2 (MGL2) and triacylglycerol lipases (TAG) found in yeast lipid droplets, thus allowing us to postulate a putative redundant function for TbAPT-L in lipolysis, along with several other predicted trypanosome ABHD proteins [66,67]. Lipases such as human ABHD16 are often sensitive to inhibition by palmostatin B [46]. Another parasite, *Toxoplasma gondii* encodes a family of four active serine hydrolases (TgASH1-4), and TgASH1 is the homologue of human APT1 with depalmitoylation activity for palmitoylated proteins which is dispensable for growth [40,54]. In contrast, TgASH2-4 hydrolyze short chain fatty acid esters and deletion causes cell defects and changes in lipid metabolites [42,68]. Therefore, lipidomics with the TbAPT-L WT, KD, and OE cell lines could be useful to determine its specific lipid substrate(s) in a future study.

Nonetheless, TbAPT-L is not a depalmitoylase, so other unidentified ABHD proteins must be responsible for depalmitoylation activity. Notably, there are two TbABHD17 homologues in the *T. brucei* genome which could be high-priority candidates. However, an unbiased approach to identify the depalmitoylase(s) in trypanosomes would be a more efficient strategy to avoid the pitfalls of making assumptions about the functions of individual proteins based on sequence similarity alone. Activity-based protein profiling (ABPP) using specific chemical probes for depalmitoylases and chemoproteomics by mass spectrometry could be useful to find candidate trypanosome depalmitoylases for validation [69]. Alternatively, performing an RNAi screen of TbABHD genes in combination with the live cell DPP-5 depalmitoylation assay would be another unbiased approach. However, there are at least fifty-five in the *T. brucei* TREU927 genome, so they would need to be prioritized for testing. Nevertheless, this highlights a very large protein fold family in trypanosomes that is relatively unstudied. Notably, the α/β-hydrolase fold has a distinctive and adaptable architecture with highly diverse catalytic (and non-catalytic) activities constituting one of the largest structurally related protein superfamilies ever characterized in eukaryotes [51]. In humans, the α/β-hydrolase domain (ABHD) family of proteins are a subset of this superfamily with 22 distinct members (ABHD1-17; 14a/b, 16a/b, 17a/b/c) many of which are implicated in lipid metabolism and signaling [18,70]. Recently, a new depalmitoylase was discovered in the mitochondrion, ABHD10 [71]. However, in trypanosomes only one crystal structure of an α/β-hydrolase fold protein has been determined for a *T. brucei* protein of unknown function (Tb927.10.10140), whose active site was highly divergent and likely catalytically inactive [56]. Identifying depalmitoylases from this ABHD family will be an important next step for understanding dynamic *S*-acylation in trypanosomes and further studies on TbAPT-L will reveal its role in lipid metabolism.

## 5. Conclusions

Palmostatin B-sensitive depalmitoylation is essential in *T. brucei* bloodstream and procyclic form parasites, but the APT-L homologue of human APT1 has no depalmitoylation activity, and instead functions as a lipase for short and medium chain acyl esters.

## Figures and Tables

**Table 1:** List of primers used in the study. All oligonucleotides were ordered from Integrated DNA Technologies (IDT).

#	Primer Name	Purpose	Sequence 5′-3′
1	Tb927.8.6390 RNAi Fwd	RNAi knockdown of TbAPT-L (***Xho*I**)	**CTCGAG**AATGGTTGGGAGAGTGTTGC
2	Tb927.8.6390 RNAi Rev	RNAi knockdown of TbAPT-L (***Hind*III**)	**AAGCTT**GATGATGTTGTCCATCGTGC
3	Tb427.8.6390 qPCR_F	qRT-PCR TbAPT-L	TTCAGTGCGGCTGAGAAAA
4	Tb427.8.6390 qPCR_R2	qRT-PCR TbAPT-L	TTCTTTGGGATGAGAGGAGTG
5	TERT Fwd	qRT-PCR TERT	GAGCGTGTGACTTCCGAAGG
6	TERT Rev	qRT-PCR TERT	AGGAACTGTCACGGAGTTTGC
7	Tb427.8.6390 pLEW79 Fwd	TET-inducible over-expression of TbAPT-L-2×myc (***Hind*III**)	**AAGCTT**ATGTTTGGCACGCCGGTTG
8	Tb427.8.6390 pLEW79 Rev	TET-inducible over-expression of TbAPT-L-2×myc (***Xba*I**)	**TCTAGA**CGATTTCGATGAAGGTCCGGG
9	YFP-TbAPT-L pLEW100v5 F	TET-inducible over-expression YFP-TbAPT-L (***Xho*I**)	GTC**CTCGAG**GGATGTTTGGCACGCCGGTTG
10	YFP-TbAPT-L pLEW100v5 R	TET-inducible over-expression YFP-TbAPT-L (***Bam*HI**) (STOP)	GTC**GGATCC**TTACGATTTCGATGAAGGTCCGG
11	TbAPT-L pET28a Fwd	IPTG-inducible TbAPT-L over-expression in *E. coli* (***Nde*I**)	**CATATG**ATGTTTGGCACGCCGGTTG
12	TbAPT-L pET28a Rev	IPTG-inducible TbAPT-L over-expression in *E. coli* (***Xho*I**) (STOP)	**CTCGAG**TTACGATTTCGATGAAGGTCCGG
13	TbAPT-L S171A Fwd	Site-directed mutagenesis TbAPT-L S171A	GTGTATGCCGGATTCGCGCAAGGTGCTGTTATTTCGCTC
14	TbAPT-L S171A Rev	Site-directed mutagenesis TbAPT-L S171A	GAAATAACAGCACCTTGCGCGAATCCGGCATACACCACG
15	TbAPT-L seq Fwd	Sequencing TbAPT-L locus	GTATTAATGGAGGAACAG
16	TbAPT-L seq Rev	Sequencing TbAPT-L locus	GATTTCGATGAAGGTCCG

## Data Availability

All relevant data are within the paper.
